# Natural course of post-COVID symptoms in adults and children

**DOI:** 10.1038/s41598-024-54397-y

**Published:** 2024-02-16

**Authors:** Aya Sugiyama, Toshiro Takafuta, Tomoki Sato, Yoshihiro Kitahara, Yayoi Yoshinaga, Kanon Abe, Chhoung Chanroth, Akuffo Golda Ataa, Zayar Phyo, Akemi Kurisu, Ko Ko, Tomoyuki Akita, Eisaku Kishita, Masao Kuwabara, Junko Tanaka

**Affiliations:** 1 Department of Epidemiology Infectious Disease Control and Prevention, Graduate School of Biomedical and Health Sciences, Hiroshima University, 1-2-3, Kasumi, Minami-ku, Hiroshima-shi, Hiroshima-ken 734-8551 Japan; 2 Hiroshima City Funairi Citizens Hospital, 14-11, Funairisaiwaicho, Naka-ku, Hiroshima-shi, Hiroshima-ken 730-0844 Japan; 3 Ministry of Health, Labor and Welfare Health Insurance Bureau Medical Economics Division, 1-2-2, Kasumigaseki, Chiyoda-ku, Tokyo-to, Tokyo 100-8916 Japan; 4 Hiroshima Prefecture Center for Disease Control and Prevention, 10-52, Motomachi, Naka-ku, Hiroshima-shi, Hiroshima-ken 730-8511 Japan

**Keywords:** Risk factors, Signs and symptoms, Viral infection

## Abstract

More than 200 million COVID-19 survivors have lasting symptoms after recovering, but the duration and related risk factors remain uncertain. This study focused on all 6551 patients diagnosed with COVID-19 at a medical institution in Hiroshima from March 2020 to July 2022. In November 2022, a questionnaire survey was conducted regarding post-COVID symptoms and their duration. The prevalence and duration of post-COVID symptoms were illustrated using the Kaplan–Meier method. Risk factors for symptoms lasting over 3 months and interfering with daily life were assessed via multivariate logistic regression. A total of 2421 survivors responded: 1391 adults, 1030 children, median age 34 years (IQR 9–55), 51·2% male, 36·7% hospitalized, median time from infection to the survey was 295 days (IQR 201–538). Upon their initial recovery, the prevalence of post-COVID symptoms was 78·4% in adults and 34·6% in children. Three months later, the rates were 47·6% and 10·8%. After over one year, they were 31·0% and 6·8%. Regarding symptoms interfere with daily life, 304 people (12.6%) experienced symptoms lasting for over three months, with independent risk factors including age, being female, diabetes mellitus, infection during the Delta period, and current smoking. There was no significant association between vaccination history and post-COVID symptoms.

## Introduction

COVID-19 is a disease caused by the severe acute respiratory syndrome coronavirus 2 (SARS-CoV-2). Since the World Health Organization (WHO) declared the COVID-19 pandemic in March 2020, the number of infections has explosively surged. As of October 2023, over 770 million people are known to have been infected, with approximately 7 million fatalities^1^. In Japan, as of May 2023, the total number of infections has been accurately recorded, with approximately 25 million people infected and around 60,000 fatalities^2^. It is known that COVID-19 symptoms can persist and result in long-term symptoms. Post COVID-19 condition, commonly known as long COVID, can affect anyone exposed to SARS-CoV-2, regardless of age or severity of original symptoms. It is defined as the continuation or development of new symptoms three months after the initial SARS-CoV-2 infection, with these symptoms lasting for at least 2 months with no other explanation^3^. The prevalence of long COVID symptoms recorded varies depending on the study subjects and methods, but it has been reported that 10–20% of individuals experience a variety of mid and long-term effects after they recover from their initial illness^4^. It is said that there are at least 200 million survivors who have experienced persistent symptoms of COVID-19^5^. However, the natural course of post-COVID symptoms and risk factors for prolonged symptoms are not yet fully understood. This study aimed to explore the natural course of post-COVID symptoms and reveal the prevalence and duration of each symptom, both in adults and children.

## Methods

### Study design and participants

This study is a retrospective cohort study conducted in collaboration with one of the Designated Medical Institutions for Class II Infectious Disease in Hiroshima, which also serves as a hub for pediatric emergency medical care. Contact information for all patients (6551 individuals) diagnosed with COVID-19 either through a positive SARS-CoV-2 polymerase chain reaction test or antigen rapid diagnostic test from March 2020 to the end of July 2022 was extracted from the medical institution's records. The study subjects included both individuals who were hospitalized and those who were not. A request for cooperation in the survey and an anonymous self-administered questionnaire, was mailed to all subjects irrespective of the SARS-CoV-2 variant they had contracted. The study examined the presence or absence, types, duration, and impact on daily life of symptoms that emerged from COVID-19 infection and persisted even after the initial recovery. Responses were collected via mail or through a dedicated internet website.

Regarding children, defined as under the age of 18, responses were provided by their parents or the children themselves. We also collected information on the severity of COVID-19, the setting for recovery at the time of infection, pre-infection vaccination history, past medical history, etc., from medical records. Notably, during this study period, the vaccines used in Japan were limited to the BNT162b2(Pfizer) and mRNA-1273(Moderna). The survey was conducted from November 2022 to March 2023. Disease severity was classified into four categories based on the need for oxygen support: mild (i.e., no need for supplemental oxygen), moderate (i.e., needed for supplemental oxygen), severe (i.e., non-invasive mechanical ventilation use), and critical (i.e., invasive mechanical ventilation use)^6^. For the epidemic periods, we categorized them as follows based on the dynamics of major variants in Hiroshima as conducted in a previous study that conducted genome analysis for confirmed COVID-19 cases^7^.

- Wild-type period: patients with disease onset between March 2020 and February 2021
- Alpha period: patients with disease onset between March and June 2021
- Delta period: patients with disease onset between July 2021 and November 2021
- Omicron period: patients with disease onset between December 2021 and July 2022

### Statistical analysis

To provide context for our analysis of post-COVID symptoms, the time period and criteria used in our study, outlined as follows:

- The point of initial recovery means the end of the patient’s quarantine period.
- The observation period is from the point of initial recovery to the disappearance of post-COVID symptoms of each patient.
- Data were considered censored if symptoms persisted beyond the time of the survey..

We calculated the prevalence of post-COVID symptoms at the initial recovery point, categorized by symptom type and for both adults and children. Using the Kaplan–Meier method, the prevalence of post-COVID symptoms was calculated at different time points after the initial recovery. We calculated the prevalence of post-COVID symptoms regardless of their severity, as well as the prevalence of post-COVID symptoms that interfere with daily life for both adults and children.

We referred to the Comprehensive Survey of Living Conditions data as a control to compare the symptoms prevalence within the general population and COVID-19 survivors from our current study. This national survey is a livelihood conditions survey in Japan, which included approximately 277,000 randomly selected households^8^. By using this reference data, we calculated the prevalence of various symptoms in the general population matched for age with the target COVID-19 survivors' population of this study.

We conducted univariate and multivariate analyses to identify risk factors associated with post-COVID symptoms persisting for more than three months and interfering with daily life. We used the following covariates: sex, age, the epidemic period (Wild-type, Alpha, Delta, Omicron), illness severity at the time of infection (Mild vs Moderate/Severe), pre-infection vaccination history (unvaccinated, 1st/2nd dose, 3rd/4th dose), current smoking, current drinking, diabetes mellitus, hypertension, and chronic renal diseases. In the univariate analysis, we performed the chi-square test and applied the Bonferroni correction when there were three or more categories. In the multivariate analysis, we employed a multivariate logistic regression model to calculate the adjusted odds ratios (AOR) and their corresponding confidence intervals. The stepwise method was used to select variables (p < 0·25).The variables excluded by the stepwise method are shown with the AOR as blank as shown in Fig. 2.

The statistical analyses were performed using JMP® Version 14 (SAS Institute Japan, Ltd., Tokyo) and p < 0·05 was considered statistically significant.

### Ethics declarations

This study was approved by the Ethics Committee of Hiroshima University (Approval No. E-2122) and conducted according to the Helsinki Declaration. Furthermore, written informed consent was obtained from each patient before any study procedure. For children, informed consent was taken from their parents or guardians.

## Results

Out of all 6551 patients (3748 adults and 2803 children) who had visited the target medical institutions and were diagnosed with COVID-19 from March 2020 to July 2022, responses were obtained from 2421 individuals (1391 adults and 1030 children), resulting in a response rate of 37.0%. The median age of the 2421 respondents was 34 years (interquartile range, IQR 9–55 years, range 0–95 years), and 51.2% of them were self-reported male (Table 1). The time of infection was as follows: the Wild-type period (April 2020–February 2021) accounted for 16.8%, the Alpha variant period (March 2021–June 2021) accounted for 13.2%, the Delta variant period (July 2021–November 2021) accounted for 13.1%, and the Omicron variant period (December 2021–July 2022) accounted for 56.9%. The median duration from infection to the survey was 295 days (IQR 201–538 days, range 96–964 days). The severity of COVID-19 was distributed as follows: asymptomatic 1.5%, mild 89.3%, moderate 7.1%, severe 1.9%, and critical 0.1%. The settings for recovery at the time of infection were as follows: at home 52.5%, in a hotel 10.9%, and at hospital 36.7%. Pre-infection vaccination history was as follows: not vaccinated 72.2%, received 1–2 doses 17.5%, received three doses 3.6%, and received four doses 0.1%. Among children, 89.6% were infected during the Omicron variant epidemic period (December 2021–July 2022), and there were no severe cases.

**Table 1:** Patients’ characteristics by epidemic period.

N		Total		Adults		Children	
		2421		1391		1030	
Age	Mean (SD), y	33·4 (25·6)		52·6(16·0)		7·5(4·4)	
	Median (IQR), y	34 (9–55)		64(41–64)		7(4–11)	
Sex	Female	1181	48.8%	727	52.3%	454	44.1%
	Male	1240	51.2%	664	47.7%	576	55.9%
Severity of COVID-19	Asymptomatic	37	1.5%	19	1.4%	18	1.7%
	Mild	2162	89.3%	1151	82.7%	1011	98.2%
	Moderate	173	7.2%	172	12.4%	1	0.1%
	Severe	46	1.9%	46	3.3%	0	0.0%
	Critical	3	0.1%	3	0.2%	0	0.0%
Epidemic periods	Wild-type period	406	16.8%	380	27.3%	26	2.5%
	Alpha period	320	13.2%	310	22.3%	10	1.0%
	Delta period	318	13.1%	247	17.8%	71	6.9%
	Omicron period	1377	56.9%	454	32.6%	923	89.6%
The locations for recovery at the time of infection	Home	1270	52.5%	393	28.3%	877	85.1%
	Quarantine hotel	263	10.9%	193	13.9%	70	6.8%
	Hospital	888	36.6%	805	57.9%	83	8.1%
Pre-infection vaccination history	None	1748	72.2%	834	60.0%	914	88·7%
	1st dose	75	3.1%	58	4.2%	17	1.7%
	2nd dose	348	14.4%	275	19.8%	73	7.1%
	3rd dose	86	3.5%	79	5.7%	7	0.7%
	4th dose	2	0.1%	2	0.1%	0	0.0%
	Unknown	162	6.7%	143	10.3%	19	1.8%

Severity was classified into four categories based on the need for oxygen or ventilation support; mild (i.e., no need for supplemental oxygen), moderate (i.e., needed for supplemental oxygen), severe (i.e., non-IMV use), and critical (i.e., IMV use).

### Prevalence of post-COVID symptoms over time in adults and children

**Figure 1:**
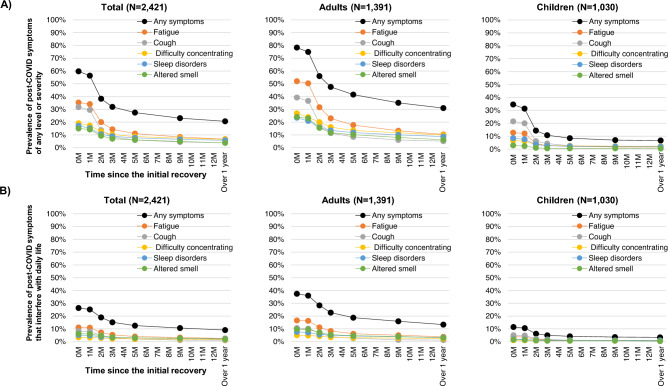
Prevalence of post-COVID symptoms over time in adults and children. Prevalence of post-COVID symptoms of any level of severity (**A**) and prevalence of post-COVID symptoms that interfere with daily life (**B**) were shown at different time points after the initial recovery in both adults and children. The symptoms tracked include any symptoms (black), fatigue (red), cough (gray), difficulty concentrating (orange), sleep disorders (blue), and altered smell (green).

The prevalence of post-COVID symptoms of any level of severity at the time of initial recovery or at the end of the quarantine period was 59.7% overall, 78.4% among adults, and 34.6% among children (Fig. 1, Table 2). In terms of symptoms, the most common symptoms among adults were fatigue (52.1%), cough (39.3%), shortness of breath (37.5%), difficulty concentrating (26.9%), altered taste (24.7%), and sleep disorders (23.5%). Among children, the prevalence of symptoms was lower compared to adults, with cough at 21.6%, fatigue at 12.8%, headache at 11.3%, sleep disorders at 8.5%, and difficulty concentrating at 6.7%. Post-COVID symptoms observed at the time of initial recovery had resolved within three months for approximately 40% of adults and approximately 70% of children. However, approximately 60% of both adults and children who had symptoms persisting beyond three months continued to experience symptoms after over one year. Post-COVID symptoms of any level of severity lasting for over one year were observed in 20·7% of the overall population, 31.0% among adults, and 6.8% among children. When comparing the prevalence of symptoms in adult survivors over one year after initial recovery to the age-matched general population, fatigue (10.4% vs 5.3%), shortness of breath (10.4% vs 1.7%), sleep disorders (8.7% vs 3.2%), memory issues (6.7% vs 3.0%), chest pain (5.1% vs 0.9%), and dizziness (4.1% vs 2.3%) were all observed at a higher frequency than in the general population. The prevalence of post-COVID symptoms in children is much lower than in adults, but for fatigue (1.8% vs 0.8%), sleep disorders (1.8% vs 0.3%), headache (1.7% vs 1.1%), dizziness (0.8% vs 0.2%), and chest pain (0.6% vs 0.1%), all of these symptoms were observed at a higher frequency than in the age-matched general pediatric population.

**Table 2:** Prevalence of Post-COVID symptoms over time in adults and children.

Symptoms of any level of severity			Any symptoms	Fatigue	Cough	Difficulty concentrating	Sleep disorders	Altered taste	Altered smell	Headache	Chest pain	Dizziness	Limb pain	Memory issues	Shortness of breath	Hair loss
Total(N = 2421)	End of the quarantine period		59.7%	35.4%	31.7%	19.2%	17.1%	15.7%	15.1%	16.2%	14.1%	9.3%	6.7%	–	–	–
	Time since infection	< 1 M	56.4%	34.1%	29.6%	17.3%	15·3%	14·9%	14·3%	15·2%	13·0%	8.7%	6.0%	–	–	–
		1–2 M	38.3%	20.0%	13.8%	12.8%	10·6%	9·3%	9·4%	7·8%	8·1%	5.3%	3.9%	–	–	–
		2–3 M	31.9%	14.4%	8.5%	10.3%	8·9%	6·5%	7·0%	6·0%	6·1%	4.5%	3.4%	–	–	–
		3–6 M	27.5%	11.1%	6.0%	8.7%	7·8%	5·0%	6·2%	4·4%	5·0%	3.8%	3.1%	–	–	–
		6–12 M	23.2%	8.4%	4.5%	7.6%	6·6%	3·8%	4·9%	3·4%	3·8%	3.1%	2.9%	–	–	–
		≥12 M	20.7%	6.7%	4.0%	6.5%	5·8%	2·7%	3·8%	3·1%	3·2%	2.7%	2.4%	–	–	–
Age-matched general population prevalence, all age			–	3·5%	4·8%	–	2·0%	–	–	3·1%	0·5%	1.5%	3.6%	–	–	–
Adults(N = 1391)	End of the quarantine period		78.4%	52.1%	39.3%	26.9%	23·5%	24·7%	24·1%	22·5%	22·7%	14.2%	9.9%	15·1%	37·5%	21·3%
	Time since infection	< 1 M	74·9%	50·4%	36.7%	23.9%	21·0%	23·7%	23·0%	21·1%	20·9%	13.4%	9.0%	12·9%	35·3%	19·2%
		1–2 M	56.0%	31·6%	19.3%	20.0%	15·7%	15·3%	15·5%	11·3%	13·0%	8.1%	6.3%	10·7%	23·4%	16·1%
		2–3 M	47·6%	23·0%	11.5%	16.1%	13·5%	10·6%	11·7%	8·6%	10·0%	7·0%	5·5%	9·4%	17·7%	14·0%
		3–6 M	41·6%	17·8%	8·5%	13·6%	11·9%	8·1%	10·2%	6·3%	8·1%	6·0%	5·0%	8·6%	14·4%	11·9%
		6–12 M	35·2%	13·3%	6·1%	11·7%	10·1%	5·9%	8·1%	4·6%	6·1%	4·9%	4·7%	7·7%	11·9%	8·2%
		≥12 M	31·0%	10·4%	5·2%	10.0%	8·7%	4·2%	6·2%	4·2%	5·1%	4·1%	3.8%	6·7%	10·4%	5·0%
Age-matched general population prevalence, adults			–	5·3%	4·6%	–	3·2%	–	–	4·4%	0·9%	2·3%	5.9%	3·0%	1·7%	–
Children(N = 1030)	End of the quarantine period		34·6%	12·8%	21·6%	6·7%	8·5%	3·4%	2·9%	11·3%	2·5%	2·5%	2.3%	–	–	–
	Time since infection	< 1 M	31·4%	12·1%	20.0%	6·2%	7·7%	3·1%	2·4%	7·1%	2·3%	2·3%	1.9%	–	–	–
		1–2 M	14·4%	4·4%	6·2%	3·5%	3·6%	1·3%	1·1%	2·9%	1·4%	1·5%	0.7%	–	–	–
		2–3 M	10·8%	2·8%	4·4%	2·9%	2·7%	1·0%	0·7%	2·2%	0·8%	1·3%	0·6%	–	–	–
		3–6 M	8·5%	2·1%	2·6%	2·5%	2·2%	1·0%	0·7%	1·8%	0·7%	1·1%	0·5%	–	–	–
		6–12 M	7·0%	1·8%	2·3%	2·2%	1·9%	1·0%	0·7%	1·7%	0·6%	0·8%	0·5%	–	–	–
		≥12 M	6·8%	1·8%	2·3%	2·1%	1·8%	0·7%	0·5%	1·7%	0·6%	0·8%	0·5%	–	–	–
Age-matched general population prevalence, children			–	0·8%	5·3%	–	0·3%	–	–	1·1%	0·1%	0·2%	0·5%	–	–	–
Symptoms that interfere with daily life			Any symptoms	Fatigue	Cough	Difficulty concentrating	Sleep disorders	Altered taste	Altered smell	Headache	Chest pain	Dizziness	Limb pain	Memory issues	Shortness of breath	Hair loss
Total(N = 2421)	End of the quarantine period		26·4%	11·2%	8·3%	3·4%	5·2%	5·8%	6·2%	4·9%	3·5%	3·4%	1·9%	–	–	–
	Time since infection	< 1 M	25·2%	11·0%	8·0%	3·2%	4·8%	5·74%	6·0%	4·8%	3·4%	3·4%	1·7%	–	–	–
		1–2 M	19·0%	7·2%	4·9%	2·9%	3·7%	4·17%	4·6%	2·8%	2·0%	2·2%	1·2%	–	–	–
		2–3 M	15·2%	5·4%	3·0%	2·4%	3·3%	2·9%	3·4%	2·4%	1·5%	2·0%	1·0%	–	–	–
		3-6 M	12·6%	4·0%	2·0%	2·1%	2·9%	2·1%	3·0%	1·8%	1·2%	1·5%	1·0%	–	–	–
		6–12 M	10·7%	3·3%	1·2%	2·0%	2·6%	1·7%	2·6%	1·5%	1·0%	1·3%	0·8%	–	–	–
		≥12 M	9·1%	2·6%	1·1%	1·6%	2·3%	1·2%	1·9%	1·4%	0·8%	1·0%	0·5%	–	–	–
Adults(N = 1391)	End of the quarantine period		37·5%	16·6%	10·7%	5·0%	7·6%	9·2%	9·7%	6·8%	5·6%	5·0%	3·0%	3·3%	9·6%	4·1%
	Time since infection	< 1 M	36·0%	16·3%	10·3%	4·8%	7·1%	9·1%	9·5%	6·7%	5·5%	4·8%	2·7%	3·1%	9·3%	3·9%
		1–2 M	28·4%	11·2%	6·9%	4·3%	5·6%	6·7%	7·3%	3·8%	3·4%	3·0%	2·0%	2·8%	7·0%	3·8%
		2–3 M	22·7%	8·3%	4·0%	3·5%	5·0%	4·7%	5·5%	3·1%	2·5%	2·6%	1·7%	2·7%	5·1%	3·6%
		3–6 M	18·8%	6·2%	2·7%	3·0%	4·4%	3·3%	4·7%	2·4%	1·9%	2·0%	1·7%	2·5%	4·0%	3·2%
		6–12 M	16·0%	5·0%	1·4%	2·8%	3·8%	2·7%	4·0%	1·9%	1·5%	1·8%	1·4%	2·3%	3·2%	2·4%
		≥12 M	13·4%	3·9%	1·2%	2·3%	3·2%	1·9%	3·0%	1·7%	1·2%	1·3%	0·9%	1·9%	2·8%	1·6%
Children(N = 1030)	End of the quarantine period		11·5%	3·8%	5·1%	1·2%	1·8%	1·3%	1·6%	2·3%	0·7%	1·4%	0·4%	–	–	–
	Time since infection	< 1 M	10·6%	3·7%	4·9%	1·1%	1·8%	1·2%	1·4%	1·5%	0·7%	1·4%	0·3%	–	–	–
		1–2 M	6·2%	1·8%	2·2%	0·9%	1·2%	0·8%	1·0%	1·5%	0·2%	1·1%	0·1%	–	–	–
		2–3 M	5·0%	1·4%	1·8%	0·9%	1·1%	0·5%	0·7%	1·1%	0·2%	1·0%	0·1%	–	–	–
		3–6 M	4·2%	1·1%	1·2%	0·9%	1·0%	0·5%	0·7%	1·0%	0·2%	0·8%	0·1%	–	–	–
		6–12 M	3·6%	0·9%	1·0%	0·9%	1·0%	0·5%	0·7%	1·0%	0·2%	0·6%	0·1%	–	–	–
		≥12 M	3·4%	0·9%	1·0%	0·8%	1·0%	0·2%	0·5%	1·0%	0·2%	0·6%	0·1%	–	–	–

National survey targeting approximately 277,000 randomly selected households.

*Comprehensive survey of living conditions.

The prevalence of post-COVID symptoms that interfere with daily life was 26.4% overall, 37.5% among adults, and 11.5% among children at the time of initial recovery. One year or more after the initial recovery, the prevalence of post-COVID symptoms that interfere with daily life was 9.1% overall, 13.4% among adults, and 3.4% among children. The prevalence of symptoms that interfere with daily life persisting for over one year was highest among adults for fatigue (3.9%), followed by sleep disorders (3.2%), altered smell (3.0%), and shortness of breath (2.8%). Among children, cough, sleep disorders, and headache were all at 1%.

### Risk factors for having post-COVID symptoms that interfere with daily life for over three months

**Figure 2:**
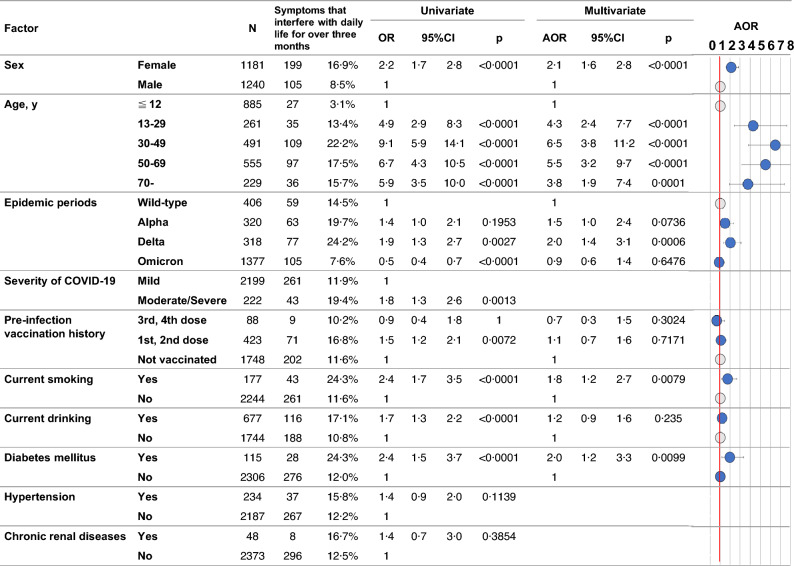
Univariate and multivariate analyses to identify risk factors associated with post-COVID symptoms that interfere with daily life for over three months. The response variable was the presence or absence of post-COVID symptoms that interfere with daily life for over three months. In the univariate analysis, the chi-square test was applied with Bonferroni correction for comparisons involving three or more groups. In the multivariate logistic regression analysis, the stepwise variable selection method was used to identify the relevant explanatory variables from a set including sex, age, the epidemic period, severity of COVID-19 at the time of infection, pre-infection vaccination history, current smoking, current drinking, diabetes mellitus, hypertension, and chronic renal diseases. N = 2421, R^2^ = 0.1267, Model p < 0.0001. The variables excluded by the stepwise method are shown with the AOR as blank. *OR* odds ratio, *CI* confidence interval, *AOR* adjusted odds ratio, *COVID-19* novel coronavirus disease 2019.

There were 304 individuals (12.6%) who had post-COVID symptoms that interfered with daily life for over three months. According to the results of the multivariate logistic regression analysis, sex, age, the epidemic period of infection, current smoking, and diabetes mellitus were identified as independent risk factors for post-COVID symptoms that interfere with daily life for over 3 months (Fig. 2). Females are 2.1 times (95% confidence interval: 1.6–2.8) at higher risk than males. Compared to those under 12 years old, the risk is 6.5 (3.8–11.2) times higher for those aged 30–49, 5.5 (3.2–9.7) times higher for those aged 50–69, 4.3 (2.4–7.7) times higher for those aged 13–29, and 3.8 (1.9–7.4) times higher for those aged 70 and over. Furthermore, in terms of the epidemic periods, individuals infected during the Delta variant period had a 2.0 (1.4–3.1) times higher risk compared to those infected during the Wild-type virus period. However, there was no significant difference between those infected during the Omicron or Alpha variant periods and Wild-type virus period. Additionally, those who currently smoke have a 1.8 (1.2–2.7) times higher risk, and those with diabetes mellitus have a 2.0 (1.2–3.3) times higher risk. On the other hand, pre-infection vaccination history, current drinking habits, and the severity of COVID-19 at the time of infection were not independent factors significantly associated with post-COVID symptoms that interfere with daily life for over three months.

## Discussion

This study aimed to elucidate the natural course of post-COVID symptoms and revealed the frequency and duration of each symptom, both in adults and children. At the point of initial recovery, 78·4% of adults still had symptoms when including mild ones, while in children, the prevalence was less than half, specifically 34·6% in comparison to adults. It has been previously reported that in children, both the severity at the time of infection and the frequency of post-COVID symptoms are lower compared to adults^9^. Regarding symptoms, fatigue, cough, and shortness of breath were most common among adults. This result is consistent with previous reports^10–15^. In children, cough was the most common complaint, followed by fatigue. Symptoms in children have been reported to be similar to those in adults, including fatigue, shortness of breath, and headaches^16^. There are few reports showing changes in the prevalence of post-COVID symptoms over time^17,18^. In this study, it was revealed that post-COVID symptoms disappear within three months in approximately 40% of adults and 70% of children. However, if these symptoms persist for over three months, about 60% of individuals continue to experience them for over a year. As a result of a multivariate analysis of risk factors for post-COVID symptoms that interfere with daily life for more than three months, sex, age, the epidemic period of infection, current smoking, and diabetes mellitus were independently significant factors. While older age has often been considered a risk factor for post-COVID symptoms^19,20^, the results of this study indicated that individuals in their working age, particularly those between 30 and 60, are at high risk for post-COVID symptoms that interfere with daily life for more than three months. The declining health of the working-age population could have broader societal implications. When compared by sex, females are twice as likely to be at risk. This is consistent with previous reports^12,19,20^. The higher risk observed in females is believed to be linked to their more robust immune response, which can be influenced by hormonal factors and other variables^21^. Diabetes mellitus and smoking history have also been reported to be risk factors for post-COVID symptoms^11,12,22^.

Regarding variant strains, it has been reported that the characteristics of post-COVID symptoms can differ depending on the specific variant strain^23^. In this study, infection with the Delta variant was identified as a risk factor for post-COVID symptoms that persist for more than three months and interfere with daily life, whereas infection with the Omicron variant did not show a significant difference compared to the Wild type. It has been reported that during the Omicron variant period, the risk of post-COVID symptoms decreased compared to the Delta variant period^11,24^. There have been reports both supporting and refuting the effectiveness of vaccines in preventing post-COVID symptoms^25–30^. The WHO is currently withholding judgment on this issue^31^. Our results did not show a preventive effect of the vaccine. As a mechanism for the preventive effect of vaccines against post-COVID symptoms, it has been pointed out that there may be an indirect effect by preventing the severity of the disease^32^. The aspect of newer vaccine variant and booster dose effects should be exploed in the future study. The results may have been influenced by the fact that the study population primarily consisted of individuals with few severe cases.

This study has several limitations. First, post-COVID symptoms were self-reported, and an assessment based on objective indicators was not performed. However, the main symptoms, such as fatigue, cannot be measured using objective indicators. Mixed-methods approach, combining self-reports with objective clinical assessments will give more comprehensive results. Second, although we asked about symptoms that appeared and persisted due to COVID-19 infection, we cannot exclude the possibility that these include symptoms that appeared regardless of whether people were infected with COVID-19. However, a comparison with the prevalence in the general population, obtained from the Comprehensive Survey of Living Conditions, was made to demonstrate the risk of symptoms among COVID-19 survivors. Third, the duration of symptoms was self-reported at the time of the survey, so there is a possibility of recall bias, which could result in both underestimation and overestimation. Fourth, although we planned to survey all patients within the period, the response rate was 37.0%. Prevalence may be overestimated due to self-selection bias. We can assume that those who did not respond might have less or no symptoms compared to who responded.

However, in terms of the prevalence of post COVID-19 condition or long COVID, most reports have focused on the prevalence at 3 or 6 months^17,18^. This study, including both adults and children, observed data over a period of more than two and a half years, making it valuable for this field. We recognize the necessity of extended follow-up periods to gain a comprehensive insight into the long-term impacts of COVID-19. Further research with longer follow-up to address this need is desired. Fifth, this study only targeted Japanese people, and comparisons with other ethnic groups were not possible because the population dynamic of Hiroshima city has very few foreign nationals. There may be an influence on social factors specific to Japan. Sixth, even though our study site is among the few major hospitals which accepted Covid 19 patients during the pandemic, it lacked the capacity to admit critically ill patients but rather focused on primarily treating patients with mild to moderate symptoms. Therefore, the analysis regarding the impact of the most severe cases, such as those requiring mechanical ventilation, is limited. Such analysis should be further investigated in future studies that include ICU patients. Additionally, the Ministry of Health Labor and Welfare (MHLW) Japan data showed that the proportion of severe COVID-19 cases in Hiroshima is estimated at most 2%, so the proportion of severe COVID-19 in our study is comparable^33^. Finally, our study couldn’t analyse lifestyle or genetic predispositions which could provide detailed risk profile. The strength of this study lies in the inclusion of all cases during the study period, encompassing both children and adults. This inclusive approach considered not only hospitalized patients but also those receiving care at home, making the data collected more representative of the general population. The results of this study will help improve our understanding of the natural course of post-COVID symptoms, which is currently not well comprehended.

## Data Availability

Regarding sharing anonymized data, it is necessary to make a direct email request to the corresponding author (jun-tanaka@hiroshima-u.ac.jp). After the evaluation of the request's content and its acceptance, data sharing will be feasible.
